# Re-description of *Balitorayingjiangensis* (Teleostei, Cypriniformes, Balitoridae) from the upper Irrawaddy River, south-western China

**DOI:** 10.3897/BDJ.13.e153915

**Published:** 2025-07-11

**Authors:** Haotian Lei, Xinrui Pu, Yinmin Geng, Yuyang Zeng, Shuwei Liu, Xiaoyong Chen

**Affiliations:** 1 Southeast Asia Biodiversity Research Institute, Chinese Academy of Sciences, Yezin, Nay Pyi Taw, Myanmar Southeast Asia Biodiversity Research Institute, Chinese Academy of Sciences Yezin, Nay Pyi Taw Myanmar; 2 College of Plant Protection, China Agricultral University, Beijing, China College of Plant Protection, China Agricultral University Beijing China; 3 Southeast Asia Biodiversity Research Institute, Chinese Academy of Sciences, Yezin, Myanmar Southeast Asia Biodiversity Research Institute, Chinese Academy of Sciences Yezin Myanmar; 4 Yunnan Agricultural University, Kunming, China Yunnan Agricultural University Kunming China; 5 Southeast Asia Biodiversity Research Institute Chinese Academy of Sciences, Yezin, Nay Pyi Taw, Myanmar Southeast Asia Biodiversity Research Institute Chinese Academy of Sciences Yezin, Nay Pyi Taw Myanmar; 6 State Key Laboratory of Genetic Resources and Evolution & Yunnan Key Laboratory of Biodiversity and Ecological Conservation of Gaoligong Mountain, Kunming Institute of Zoology, Chinese Academy of Sciences, Kunming, China State Key Laboratory of Genetic Resources and Evolution & Yunnan Key Laboratory of Biodiversity and Ecological Conservation of Gaoligong Mountain, Kunming Institute of Zoology, Chinese Academy of Sciences Kunming China; 7 Yunnan International Joint Laboratory of Southeast Asia Biodiversity Conservation, Mengla, China Yunnan International Joint Laboratory of Southeast Asia Biodiversity Conservation Mengla China; 8 University of Chinese Academy of Sciences, Beijing, China University of Chinese Academy of Sciences Beijing China; 9 Southeast Asia Biodiversity Research Institute, Chinese Academy of Sciences, Yezin, Nay Pyi Taw, China Southeast Asia Biodiversity Research Institute, Chinese Academy of Sciences Yezin, Nay Pyi Taw China

**Keywords:** Balitoridae, *Balitora*, biodiversity, phylogeny, morphology, freshwater fish, Irrawaddy River

## Abstract

**Background:**

The genus *Balitora* Grey, 1830 (Teleostei, Cypriniformes, Balitoridae) includes 20 species, with two known species distributed in the Salween River and one in the Irrawaddy River.

**New information:**

A *Balitora* species from the Irrawaddy River, *Balitorayingjiangensis*, which was previously mistakenly classified as *Hemimyzon*, is re-described through examination of type specimens and collection of topotypic specimens from the Jieyanghe River, a tributary of the Irrawaddy River in Nabang Town, Yingjiang County, Yunnan Province, China. *Balitorayingjiangensis* differs from all congeners by the following combination of characters: one pair of maxillary barbels; short predorsal length, 43.48%-36.13% SL; slender caudal peduncle, length of caudal peduncle 3.71-4.47 times of depth; shallow caudal peduncle, height of which 4.14%-4.84% SL. Molecular phylogenetic analysis supports the validity of this species and suggests a wide distribution throughout the Chinese and Burmese Irrawaddy. *Balitoraburmanica* is not distributed in the Irrawaddy River, previous records of which from Chinese literature are mistaken records of *B.yingjiangensis*.

## Introduction

The hill-stream loach genus *Balitora* Grey, 1830 is widely distributed in the torrential habitats of south and south-eastern Asia, currently represented by 20 species. This taxon is characterised by a series of rheophilic adaptations, namely horizontally situated and enlarged pair fins with adhesive pads covered by unculi (Roberts 1982) and a strongly depressed body profile, enabling them to cling on to rocky substrates of torrential rivers and feed on aquatic invertebrates and algae.

This genus was established by Grey as a member of Cobitidae with *B.brucei* as type species (Gray 1830) and currently placed in family Balitoridae (Fricke et al. 2025), differentiating from closely-related genera, namely *Hemimyzon* Regan 1911 and *Sinohomaloptera* Fang 1935, by a combination of a papillated mouth with one pair of maxillary barbels and two simple pelvic fin rays (Chen 1978). Kottelat and Chu (1988) and Kottelat (1988) reviewed *Balitora* and considered *Sinohomaloptera* to be a synonym of which, with *Balitora* possessing one or two pairs of maxillary barbels. Based on three or more simple pelvic fin rays, *B.pengi*, *B.tchangi*, *B.nujiangensis* and *B.elongata* were placed in *Hemimyzon*, while species with two branched pelvic fin rays were placed in *Balitora*, namely *B.lancangjiangensis* (Zheng 1980), *B.kwangsiensis* (Fang 1930) and *B.longibarbata* Chen, 1982 (Zheng et al. 1982, Kottelat and Chu 1988). A controversy on the validity of *Sinohomaloptera* took place amongst Chinese researchers, with some supporting the hypothesis of Kottelat and Chu (1988). (Chen 1990) and others continued considering *Sinohomaloptera* valid (Chen and Tang 2000, Jiang et al. 2016). The adoption of phylogeny in the taxonomy of Balitorinae revealed a complex intergeneric relationship and possible non-monophyly of *Balitora* (Liu et al. 2012, Kumkar et al. 2016, Keskar et al. 2018, Tao et al. 2019, Shao et al. 2020). To date, a comprehensive phylogenetic analysis on Balitoridae species is yet to come.

Four species from Vietnamese literature, namely *B.haithanhi*, *B.nigrocorpa*, *B.vanlani* and *B.vanlongi* (Nguyen 2005) are considered invalid by Kottelat (Kottelat 2012, Kottelat 2013), yet only *B.nigrocorpa* is recorded as a synonym of *B.kwangsiensis* by Fricke et al. (2025) due to lack of detailed explanation and evidence in Kottelat's treatement (Kottelat 2012, Kottelat 2013). Given the fact that type specimens of these four species are confirmed lost (Endruweit 2014), all four Vietnamese species are considered invalid in this research and, therefore, excluded in the morphological analysis.

Amongst the 20 recognised species of *Balitora*, only one species, *B.burmanica* (Hora 1932) was previously recorded from the Irrawaddy River Basin. Hora (1932) described *B.burmanica*, based on specimens from the lower Salween River in Meekalan and Meetan, the upper Irrawaddy River in Myitkyina District, as well as the Shishak River (tributary of the upper Karnaphuli River) in the Chittagong Hill Tracts. However, no holotype or paratype was selected and no morphometric data were provided. Kottelat (1988) examined part of the syntypes, designated a lectotype from Meekalan and provided the first morphometric data of *B.burmanica*, thus suggesting that *B.burmanica* is distributed in both the Salween River and the Irrawaddy River. This suggestion has been accepted by scholars worldwide ever since, with records of balitorids with two simple pelvic fin rays from the Chinese Irrawaddy also classified as *B.burmanica* (Talwar and Jhingran 1991, Chen et al. 2005, Kottelat 2012, Chen 2013, Tian et al. 2022).

Yet, Kottelat (1988) did not examine any specimen from the Irrawaddy River and the sole morphological comparison of *Balitora* spp. from the Irrawaddy and Salween River was made by Chen et al. (2005), who described *B.nantingensis* from the Nanting River, a tributary of the upper Salween River, thus confirming the existence of difference between the *Balitora* species of the two Basins.

In addition, a *Hemimyzon* species, *H.yingjiangensis* (Chen 2006) was described in accordance with specimens from Menglai River, Yingjiang County, Yunnan Province, China, a tributary of the upper Irrawaddy River. Chen (2006) placed it in *Hemimyzon* for possessing three simple pelvic fin rays.

Specimens of balitorid fish were obtained from the upper and middle Irrawaddy River as well as the lower Salween River during multiple ichthyological surveys conducted from 2017 to 2024. Through comprehensive morphological and phylogenetic analysis, *H.yingjiangensis* is confirmed valid as a *Balitora* species and the sole balitorinae species of the upper Irrawaddy River in China and Myanmar, hereby re-described as *B.yingjiangensis*.

## Materials and methods

### Sample collection

All care and use of experimental animals complied with the relevant laws of the Chinese Laboratory of Animal Welfare and Ethics (GB/T 35892-2018). All specimens were euthanised immediately after capture, fixed and subsequently stored in formalin solution. Tissue samples were taken from the fins and preserved in 95% ethanol.

A total of sixteen specimens of *Balitora* spp. were obtained, one from the lower Salween River in Myanmar and fifteen from the upper Irrawaddy River in China and Myanmar, amongst which eight are topotypic specimens of *Hemimyzonyingjiangensis*. An additional two specimens of *H.nujiangensis* were collected from the upper Salween River. All specimens were preserved in 8% formalin and stored at the Kunming Zoological Museum, Kunming Institute of Zoology (KIZ), Chinese Academy of Sciences.

### Morphometric data acquisition

Morphological research on Balitorids from the Salween and Irrawaddy River was conducted through voucher specimens and literature.

Measurements were made point-to-point with digital calipers and data were recorded to an accuracy of 0.01 mm. Measurements of all specimens examined were made on the left side of specimens whenever possible. Subunits of the head are presented as percentage proportions of head length (% HL). Head length and measurements between landmarks on the body are given as percentage proportions of standard length (% SL). Detailed information of morphometric variables follows Kottelat (Kottelat 1988, Kottelat 2001). The type specimens as well as newly-collected specimens of *H.yingjiangensis* were compared with available specimens of other known members of *Balitora* from the Irrawaddy and Salween Rivers, i.e. *B.nantingensis* Chen and *B.burmanica* Hora.

Literature review consisted primarily of original descriptions of species and genera as well as results from important comprehensive studies (Gray 1830, Fang 1930, Hora 1932, Hora 1941, Zheng 1980, Zheng et al. 1982, Kottelat 1988, Chen et al. 2005, Chen 2006, Conway and Mayden 2010, Bhoite et al. 2012, Liu et al. 2012, Raghavan et al. 2013, Kumkar et al. 2016, Luo et al. 2023).

### DNA extraction, amplification, sequencing and phylogenetic analysis

Fin tissue for molecular analysis was taken from fresh specimens and preserved in 95% ethanol. Primers used in PCR amplification are listed in Table 1. The mitochondrial cytochrome b gene (Cyt *b*) and cytochrome oxidase subunit 1 (COI) gene of selected individuals was amplified and sequenced adopting the Protocols of DNA extraction and PCR amplification of Wu et al. (2024). Purification and sequencing in both forward and reverse directions were undertaken at Sangon Biotech (Shanghai, China) Co., Ltd.

The sequencing results were manually assembled using SeqMan II, and other sequences were acquired from the NCBI database. A total of 56 Cyt *b* and 40 COI gene fragments were included in the phylogenetic analysis. The voucher ID of each individual and GenBank accession nos. are given in Table 2. All selected sequences are aligned by Clustal W in MEGA 11 (Tamura et al. 2021). Two methods were used to construct the phylogenetic tree for the concatenated dataset of two genes: Maximum Likelihood (ML) and Bayesian Inference (BI). *Paracanthocobitismorreh* was used as an outgroup. ModelFinder (Kalyaanamoorthy et al. 2017) was used to select the best-fit model using Bayesian Information Criterion (BIC). All operations of ML and BI were done in PhyloSuite v.1.2.2 (Zhang et al. 2020). Node support was determined by setting 1,000 bootstrap (BS) replicates in ML and 20,000,000 generations of Markov chains, sampling every 2,000 generations to yield 10,000 trees in BI. The Tree is visualised in FigTree 1.4.4 (Rambaut 2018) and iTOL (https://itol.embl.de/). Uncorrected p-distances (1000 replicates), based on Cyt *b*, were estimated using MEGA11 (Tamura et al. 2021).

### Comparative materials

1. *Balitoranantingensis*: KIZ20026475, KIZ20026471, KIZ20026470, KIZ20026472, Mangjiu River, a tributary of Nanting River, Daxueshan Township, Yongde County, Lincang Prefecture, Yunnan Province, China.

2. *Balitoraburmanica*: KIZ2002001925, KIZ2002001933, KIZ2002009671, Mangjiu River, a tributary of Nanting River, Daxueshan Township, Yongde County, Lincang Prefecture, Yunnan Province, China; CXY20190208, Yaeboat Dam, Hpa An, Myanmar.

3. *Balitoratchangi*: WL20240228, Lancangjiang River, Weixi Lisu Autonomous County, Diqing Tibetan Autonomous Prefecture, Yunnan Province, China.

4. *Hemimyzonnujiangensis*: ZYY20230068, Nanting River, Gengma County, Lincang City, Yunnan Province, China; LSW202278, Mengnuo Township, Longling County, Baoshan City, Yunnan Province, China.

5. *Jinshaianiulanjiangensis*: LHT20240501, Niulanjiang River, Deze Township, Zhanyi District, Qujing City, Yunnan Province, China.

6. *Jinshaiaabbreviata*: PXR20240816, Dadu River, Leshan City, Sichuan Province, China.

7. *Hemimyzonmegalopseos*: PXR20231011, Nanpan River, Kaiyuan City, Honghe Prefecture, Yunnan Province, China.

8. *Hemimyzonmacroptera*: PXR20231113, Nanpan River, Kaiyuan City, Honghe Prefecture, Yunnan Province, China.

## Taxon treatments

### Balitora yingjiangensis

(Chen, 2006)

E30FDD56-55B2-5887-A659-65C003111CA8

AE32975A-ECE8-421D-B2B3-677554C4D6D3

*Hemimyzonyinjiangensis* Chen 2006 (original description).

#### Materials

- **Type status:** Holotype. **Occurrence:** occurrenceID: 04342065-F302-535F-B7B2-25620420FEE2; **Location:** locationID: KIZ1995000888; continent: Asia; waterBody: Irrawaddy River; country: China; countryCode: CN; stateProvince: Yunnan; county: Yingjiang; municipality: Jieyanghe River; verbatimCoordinateSystem: decimal degrees; decimalLatitude: 24.711469; decimalLongitude: 97.577983; **Event:** eventDate: Apr 1995
- **Type status:** Paratype. **Occurrence:** occurrenceID: 19FA9671-B453-580E-B67A-E6B1823B09B3; **Location:** locationID: KIZ1995000889; continent: Asia; waterBody: Irrawaddy River; country: China; countryCode: CN; stateProvince: Yunnan; county: Yingjiang; municipality: Jieyanghe River; verbatimCoordinateSystem: decimal degrees; decimalLatitude: 24.711469; decimalLongitude: 97.577983; **Event:** eventDate: Apr 1995
- **Type status:** Other material. **Occurrence:** occurrenceID: D44BB2D9-0128-56F5-8270-4DB110D50786; **Location:** locationID: KIZ20220256, KIZ20220255, KIZ20220257 and KIZ20220258(4); continent: Asia; waterBody: Irrawaddy River; country: China; countryCode: CN; stateProvince: Yunnan; county: Yingjiang; municipality: Jieyanghe River; verbatimCoordinateSystem: decimal degrees; decimalLatitude: 24.711469; decimalLongitude: 97.577983; **Event:** eventDate: 21 Jul 2022
- **Type status:** Other material. **Occurrence:** occurrenceID: 3AA633F0-9FB8-5EC2-ACDF-9A772269E479; **Location:** locationID: PXR20231115001, PXR20231115002(2); continent: Asia; waterBody: Irrawaddy River; country: China; countryCode: CN; stateProvince: Yunnan; county: Yingjiang; municipality: Jieyanghe River; verbatimCoordinateSystem: decimal degrees; decimalLatitude: 24.711469; decimalLongitude: 97.577983; **Event:** eventDate: 15 Nov 2023
- **Type status:** Other material. **Occurrence:** occurrenceID: 980C451B-5F1E-59F3-B80C-38D25F5654BA; **Location:** locationID: PXR20240512001, ZYY20240049(2); continent: Asia; waterBody: Irrawaddy River; country: China; countryCode: CN; stateProvince: Yunnan; county: Yingjiang; municipality: Jieyanghe River; verbatimCoordinateSystem: decimal degrees; decimalLatitude: 24.711469; decimalLongitude: 97.577983; **Event:** eventDate: 12 May 2024
- **Type status:** Other material. **Occurrence:** occurrenceID: 1CE6328F-7FF3-5BAC-AD11-30128BEAB69F; **Location:** locationID: KIZ200309246; continent: Asia; waterBody: Irrawaddy River; country: China; countryCode: CN; stateProvince: Yunnan; county: Tengchong city; municipality: Longchuanjiang River; verbatimCoordinateSystem: decimal degrees; decimalLatitude: 24.889876; decimalLongitude: 98.675866; **Event:** eventDate: 15 Sep 2003
- **Type status:** Other material. **Occurrence:** occurrenceID: 02126BC8-499F-55AE-B580-C10A856CEFD0; **Location:** locationID: KIZ2006009598, KIZ2006009536, KIZ2006009525 and KIZ2006009539(4); continent: Asia; waterBody: Irrawaddy River; country: China; countryCode: CN; stateProvince: Yunnan; county: Tengchong city; municipality: Longchuanjiang River; verbatimCoordinateSystem: decimal degrees; decimalLatitude: 24.889876; decimalLongitude: 98.675866; **Event:** eventDate: 23 Apr 2006
- **Type status:** Other material. **Occurrence:** occurrenceID: 15E5F684-ABDB-5136-97EB-CB7DD3BE2457; **Location:** locationID: KIZ20220332; continent: Asia; waterBody: Irrawaddy River; country: China; countryCode: CN; stateProvince: Yunnan; county: Ruili city; municipality: Ruili River; verbatimCoordinateSystem: decimal degrees; decimalLatitude: 24.102258; decimalLongitude: 97.995292; **Event:** eventDate: 26 Jul 2022
- **Type status:** Other material. **Occurrence:** occurrenceID: CB4368D8-40E1-54A7-802A-8056C238CC48; **Location:** locationID: QT20170109, QT20170106, QT20170108 and QT20170107(4); continent: Asia; waterBody: Irrawaddy River; country: Myanmar; countryCode: MM; stateProvince: Kachin State; county: Putao District; municipality: stream near Hkyenhpa Populated place; verbatimCoordinateSystem: decimal degrees; decimalLatitude: 27.672414; decimalLongitude: 97.384083; **Event:** eventDate: 29 Nov 2017
- **Type status:** Other material. **Occurrence:** occurrenceID: EFBE7DAD-E960-5D5A-9CA8-11721665CB9A; **Location:** locationID: CXY20150315; continent: Asia; waterBody: Irrawaddy River; country: Myanmar; countryCode: MM; stateProvince: Kachin State; county: Putao District; municipality: Chan Khaung Stream; verbatimCoordinateSystem: decimal degrees; decimalLatitude: 27.408914; decimalLongitude: 97.315847; **Event:** eventDate: 28 Dec 2015
- **Type status:** Other material. **Occurrence:** occurrenceID: 4E863060-EA7E-59D2-B880-DF0470C507DC; **Location:** locationID: CXY20190496; continent: Asia; waterBody: Irrawaddy River; country: Myanmar; countryCode: MM; stateProvince: Sagaing Region; county: Kale District; municipality: Yaw Su Village; locality: a primary tributary of Chindwin River; verbatimCoordinateSystem: decimal degrees; decimalLatitude: 23.187093; decimalLongitude: 94.270935; **Event:** eventDate: 6 Nov 2019

#### Description

Morphometric and meristic data of 11 specimens of *B.yingjiangensis* are provided in Table 3. See Fig. 3 for dorsal, lateral and ventral profile of body. Body elongated, rounded laterally and flattened ventrally, posterior portion gradually compressed from dorsal fin to caudal-fin base, with the highest point of body at dorsal-fin origin. Body covered in small scales, lateral-line scales 63-67. Each scale with a prominent longitudinal keel along centre. Lateral-line scales with two additional smaller keels along posterior edge, above and below principal longitudinal keel.

Head blunt and depressed, dorsum of head densely covered with tiny tubercles. Head length greater than head width and head width much greater than head depth. Eyes small, dorsally situated, slightly posterior of the mid-point of head, not visible from ventral view. Interorbital space wide and flat.

Mouth small, inferior, relatively shallow pre-oral groove present between rostral cap and upper lip, extending across corners of mouth. Mouth width 23.66–33.23% of head width. Rostral cape around upper lip and divided into three lobes, the median one the largest, slightly curved. Two pairs of rostral barbels, both shorter than eye diameter, outer one slightly longer than the inner one. One pair of maxillary barbels, shorter than eye diameter, situated at corner of mouth. Upper and lower jaws covered by a horny sheath. Upper and lower lips with a single row of large papillae. Lower jaw with radiating ridges on surface. Two longitudinal fleshy ridges on mid-chin (Fig. 5). Gill opening large, extending to the pectoral fin origin on the ventral surface.

Dorsal fin rays iiii, 7, pectoral fin rays ix,14, pelvic fin rays ii, 9, anal fin rays iii, 5 and principal caudal fin rays 6+7. Dorsal fin origin slightly posterior to pelvic-fin origin and nearer to tip of snout than to caudal fin base. Pectoral fins large and horizontally placed, posterior margin rounded. Tip of the pectoral fin not reaching pelvic fin origin. Pelvic fins short, not reaching or just reaching anus, posterior margin straight. Pelvic fin origin closer to snout tip than caudal fin base and closer to anal fin origin than snout tip. Anus situated approximately 4/5 distance from posterior end of the pelvic fin base to the anal fin origin. Anal fin short, origin close to the anus and far from the caudal fin base, tips of which not reaching caudal fin base when extended backwards. Caudal fin deeply forked, lower lobe longer than upper lobe. Uppermost and lowermost three or four principal caudal rays without interradial membranes and tightly associated. Caudal peduncle slender, length of which 3.71-4.47 times depth.

#### Diagnosis

*Balitorayingjiangensis* is distinguished from *B.anlongensis*, *B.longibarba*, *B.ludongensis* and *B.kwangsiensis* in possession of one pair of maxillary barbels vs. two pairs of maxillary barbels, distinguished from *B.annamica*, *B.eddsi*, *B.jalpalli*, *B.laticauda*, *B.meridonalis* and *B.mysorensis* in a shorter predorsal length, 36.1-43.5% SL vs. greater than 43.7% SL, from *B.chipkali* and *B.lancangjiangensis* in a slenderer caudal peduncle, length of caudal peduncle 3.71-4.47 times depth vs. less than 3.5 times and from *B.tchangi* from two simple pelvic fin rays vs. three simple pelvic fin rays. *B.brucei*, *B.burmanica* and *B.nantingensis* are closely-related congeneric species of *B.yingjiangensis*. *B.yingjiangensis* is differentiated from *B.brucei*, *B.burmanica* and *B.nantingensis* in a lower caudal peduncle, 4.14-4.84% SL vs. 5.10-5.80% in *B.brucei*, 5.01-5.81% in *B.burmanica* and 5.03-6.28% in *B.nantingensis*. It further differs from *B.burmanica* and *B.nantingensis* in a slenderer caudal peduncle, length of caudal peduncle 3.71-4.47 times depth vs. 3.46-3.03 times in *B.burmanica* and 2.94-2.62 times in *B.nantingensis* and a smaller head, head length 18.35-20.47% SL vs. 21.12-22.24% in *B.burmanica* and 20.54-23.10% in *B.nantingensis*. It further differs from *B.nantingensis* in a larger mouth, width of mouth 20.17-25.30% HL vs. 14.87-19.33% and larger eyes, eye diameter 14.5-20.05% HL vs. 11.19-12.89%.

#### Distribution

**Figure 1.**
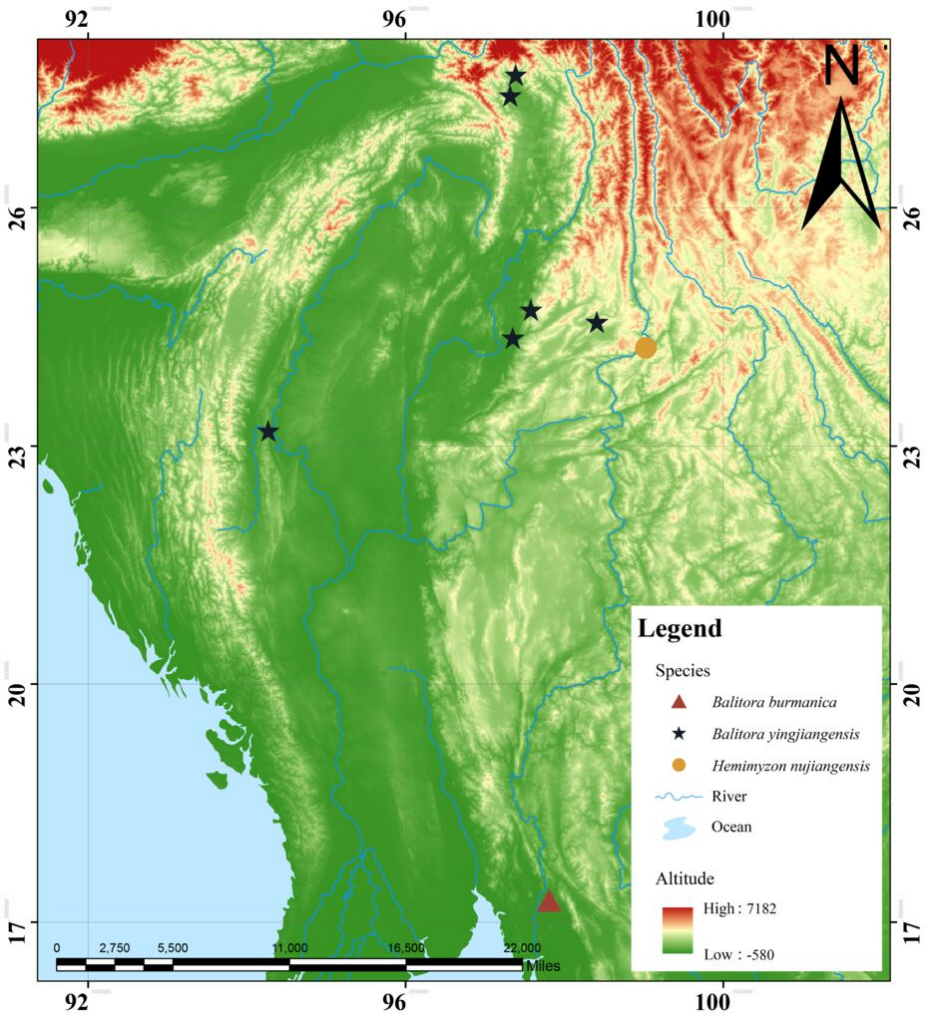
Map exhibiting sampling sites of three Balitorinae species from the Irrawaddy and Salween Rivers, *B.burmanica* (triangle) *Balitorayingjiangensis* (asterisk) and *Hemimyzonnujiangensis* (circle).

*Balitorayingjiangensis* is known from upper tributaries of the Irrawaddy River in China and Myanmar, namely the Chindwin River, Dayingjiang River, Longchuanjiang-Ruilijiang River and Menglaihe River. The type locality is in a reach of the Jieyanghe River at Nabang Town, Yingjiang County, Yunnan Province, China (Fig. 1).

#### Ecology

This species inhabits torrential rivers with clear, well oxygenated water as well as boulder and gravel substrate. Mainly found in streams and shallow rivers, rarely in the main course. Enlarged and flattened paired fins enable it to resist the current and cling on to the rocky surface while feeding on algae and aquatic invertebrates. Syntopic fish species observed together with *B.yingjiangensis* include: *Neolissochilusheterostomus*, *Garrarotundinasus*, *Psilorhynchusolliei*, *Schistura* sp., *Glyptothoraxminimaculatus*, *Pseudecheneisbrachyura* and *Exostomavinciguerrae*.

#### Colouration

In living specimens, dorsal and lateral sides of the body greyish-brown, creamy ventrally. Dorsum of head dark brown, covered with white tubercles. Medium of body with seven saddle-shaped dark blotches situated longitudinally in the dorsal profile. Sides of body with longitudinal black band along lateral line and scattered black spots. Dorsal fin rays light brown with a black bar at the middle 1/3 of rays, interradial membranes hyaline. Pectoral and pelvic fin bases brown with black blotches from dorsal view. Pectoral and pelvic fin rays dark bronze, membrane lighter, medium of which with anterior curving black stripes. Anal fin hyaline with a light black bar on proximal rays. Upper lobe of caudal fin light brown with a light black bar in the middle. Lower lobe of caudal fin with a prominent longitudinal black band, uppermost and lowermost fin rays bronze (Fig. 4).

##### Etymology

The specific epithet “*yingjiangensis*” refers to the type locality： Nabang Town, Yingjiang County, Yunnan Province, China. We propose the common English name“Yingjiang hillstream loach” and the Chinese name “Ying Jiang Pa Qiu (盈江爬鳅)".

## Identification Keys

### Key to the species of *Balitora* from the Irrawaddy and Salween River

**Table:** 

1	Caudal peduncle lower, its depth less than 5% SL	*B.yingjiangensis*
–	Caudal peduncle deep, its depth more than 5% SL	2
2	Caudal peduncle slender, length of which greater than 3 times depth	*B.burmanica*
–	Caudal peduncle thick, length of which less than 3 times depth	*B.nantingensis*

A key to the three species of *Balitora* is shown below.

## Analysis

### Molecular phylogeny

**Figure 2.**
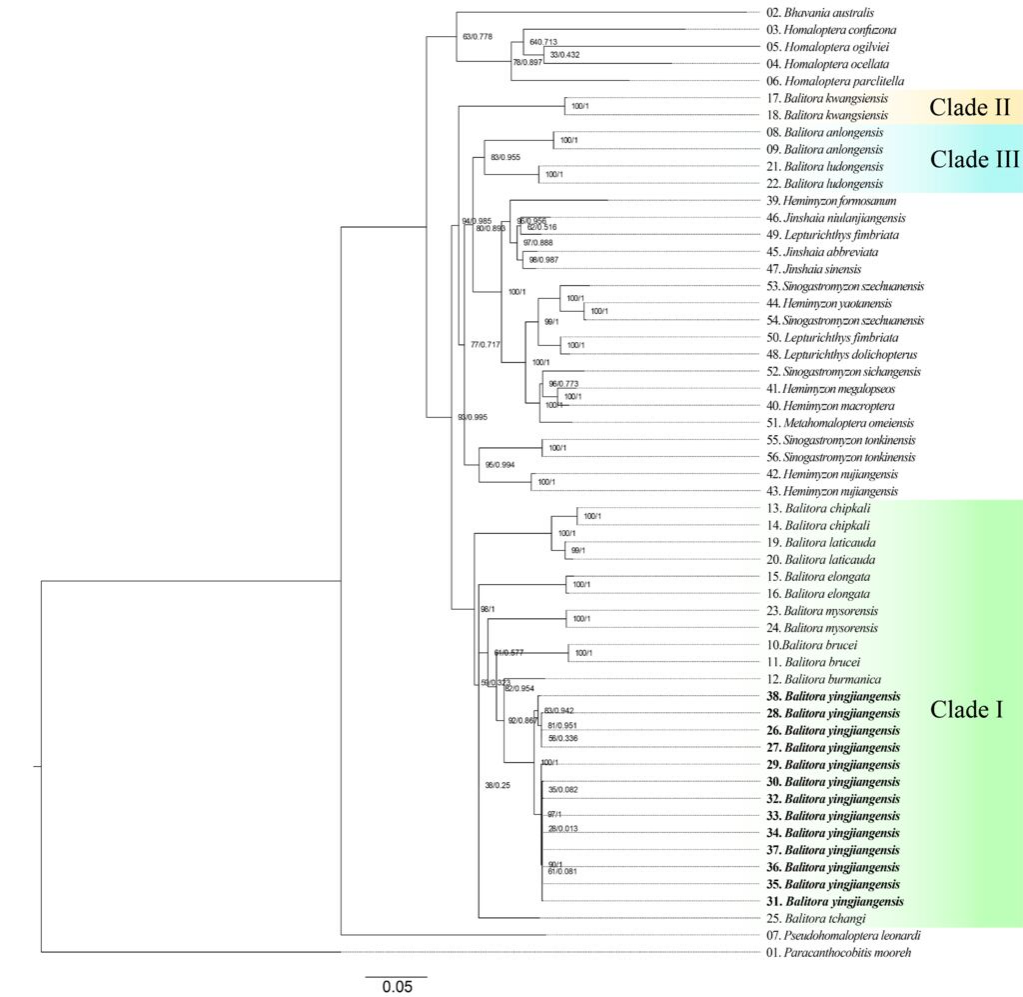
Phylogenetic tree of Balitorinae based on mitochondrial Cyt *b* and COI gene. The scale represents 0.05 nucleotide substitution at each site. Numbers beside nodes are ultrafast bootstrap (UFB) supports from Maximum Likelihood (ML) analyses/Bayesian Posterior Probabilities (BPP) from Bayesian Inference (BI) analyses.

Both phylogenetic methods (ML and BI) recovered a similar tree topology (Fig. 2), based on two mitochondrial gene fragments (Cyt *b*, COI). Although *Balitora* exhibited polyphyly, all Indian and Indochinese *Balitora* species possessing a single pair of maxillary barbels, including *B.yingjiangensis* (referred as clade I) formed a monophyly with strong support. *Balitora* species with two pairs of maxillary barbels formed two distinct monophyletic clades, namely clade II, represented only by *B.kwangsiensis* and clade III represented by *B.anlongensis* and *B.ludongensis*. Closely-related haplotypes were observed in *B.yingjiangensis* and its monophyly is strongly supported. Phylogenetic analyses supported *B.yingjiangensis* being closely related to *B.burmanica* from the Salween River and *B.brucei* from the Brahmaputra River. The interspecific variations between *B.yingjiangensis* and other *Balitora* species of clade I are estimated with minimum interspecific p-distance values, ranging from 5.61% (with *B.burmanica*) to 10.74% (*B.chipkali*) for Cyt *b*, significantly greater than maximum intraspecific p-distance of *B.yingjiangensis* (1.68%). (Table 4).

## Discussion

### Balitorayingjiangensis, the sole confirmed Balitora species of the upper Irrawaddy

The taxonomic status of *Balitora* spp. in the Irrawaddy River Basin has long been ambiguous. *Balitoraburmanica* was originally described by Hora (1932), based on specimens from three disjunct drainages - Irrawaddy, Salween and Karnaphuli Rivers - characterised by a narrower body, longer head and darker pigmentation compared to *B.brucei*. However, Hora's description lacked morphometric analyses and type designation and no holotype or type locality was designated. Subsequent work by Silas (1953) listed three specimens from Meekalan (ZSI F 11034/1) as "Type (original statement)" without any further explanation of whether these specimens were considered part of syntypes or is this a lectotype designation. Kottelat's (Kottelat 1988) revision designated a lectotype and eight paralectotypes from the same locality and referred to Slias' reference of the three specimens as [Holo]type "not correct" as the holotype should be unique. This statement is inappropriate as Silas (1953) did not provide explanation about the substitution of this "Type" and it seems arbitrary to define it as holotype without any evidence or demonstration. Despite this, we follow the lectotype designation of Kottelat (1988) in this study as the intention of listing "Type" in Silas (1953) could not be confirmed, but with reservations for adjustments of the lectotype with sufficient evidence. The type locality of *B.burmanica* is thus Meekalan (Salween River). Kottelat (1988) revised *B.burmanica* in exclusive accordance to Salween specimens and no Irrawaddy representatives were examined. This left the Irrawaddy records of *B.burmanica* supported only by Hora's brief mention of a single specimen from Myitkyina (ZSI F 10886/1) without diagnostic details and this conclusion has been followed by researchers worldwide ever since (Talwar and Jhingran 1991, Chen et al. 2005, Kottelat 2012, Chen 2013, Tian et al. 2022).

**Figure 3.**
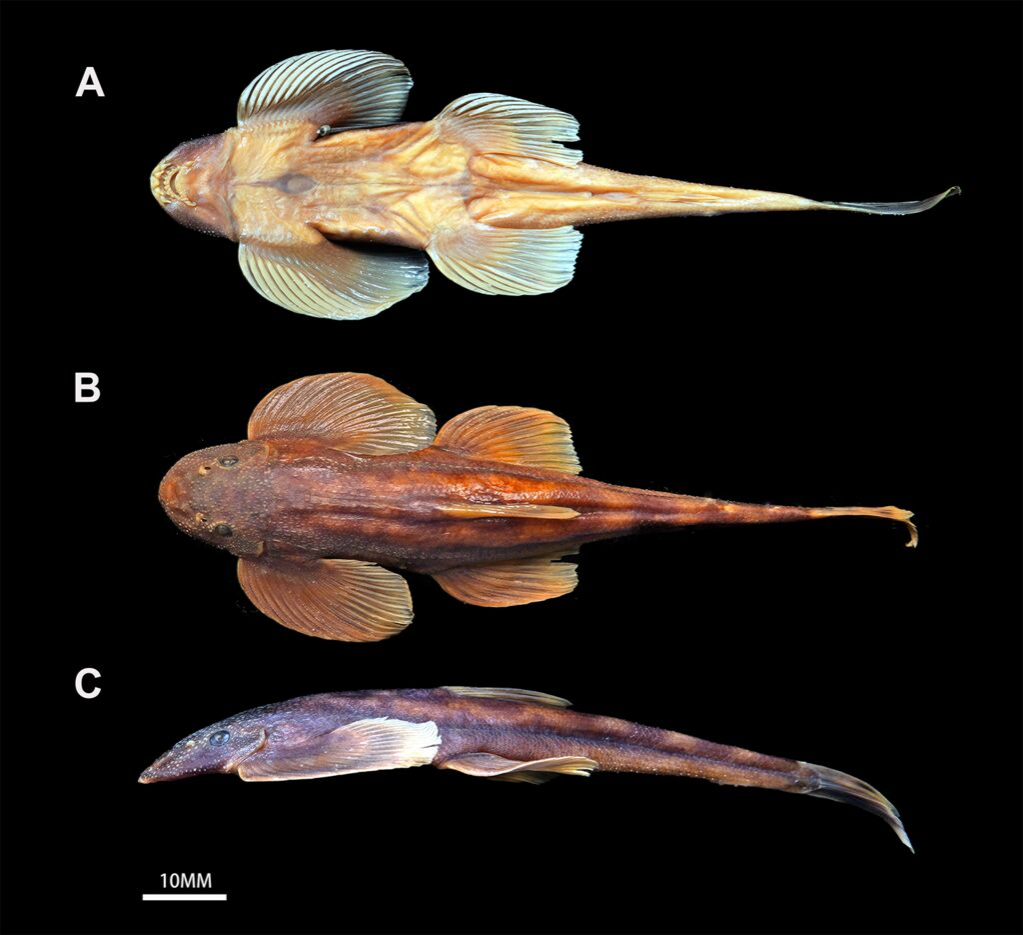
Morphological characteristics of *Balitorayingjiangensis* (PXR20231115002); colouration in preservation; **A** ventral view; **B** dorsal view; **C** lateral view.

**Figure 4.**
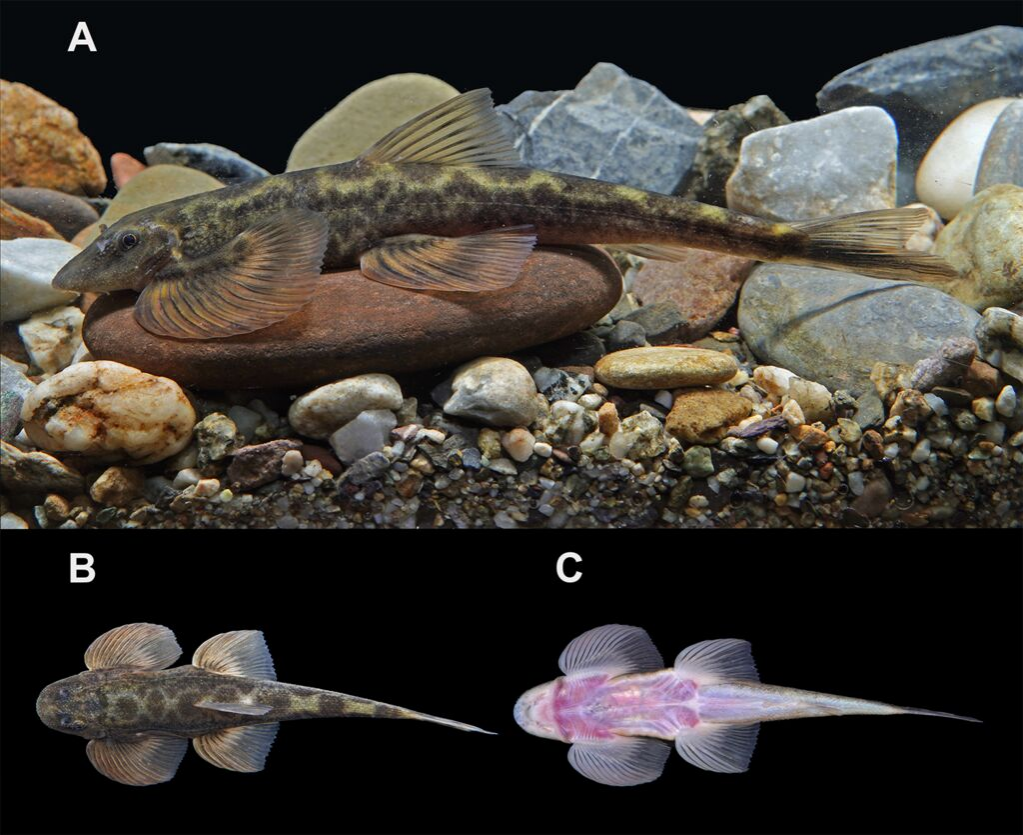
Morphological characteristics of *Balitorayingjiangensis* (PXR20231115002); colouration in life; **A** lateral view; **B** dorsal view; **C** ventral view.

**Figure 5.**
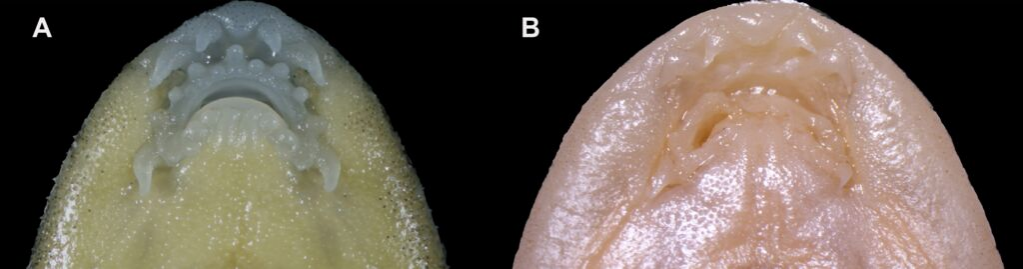
Oral morphology of *Balitorayingjiangensis*; **A** PXR20231115003; **B** KIZ1995000888 (Holotype).

**Figure 6.**
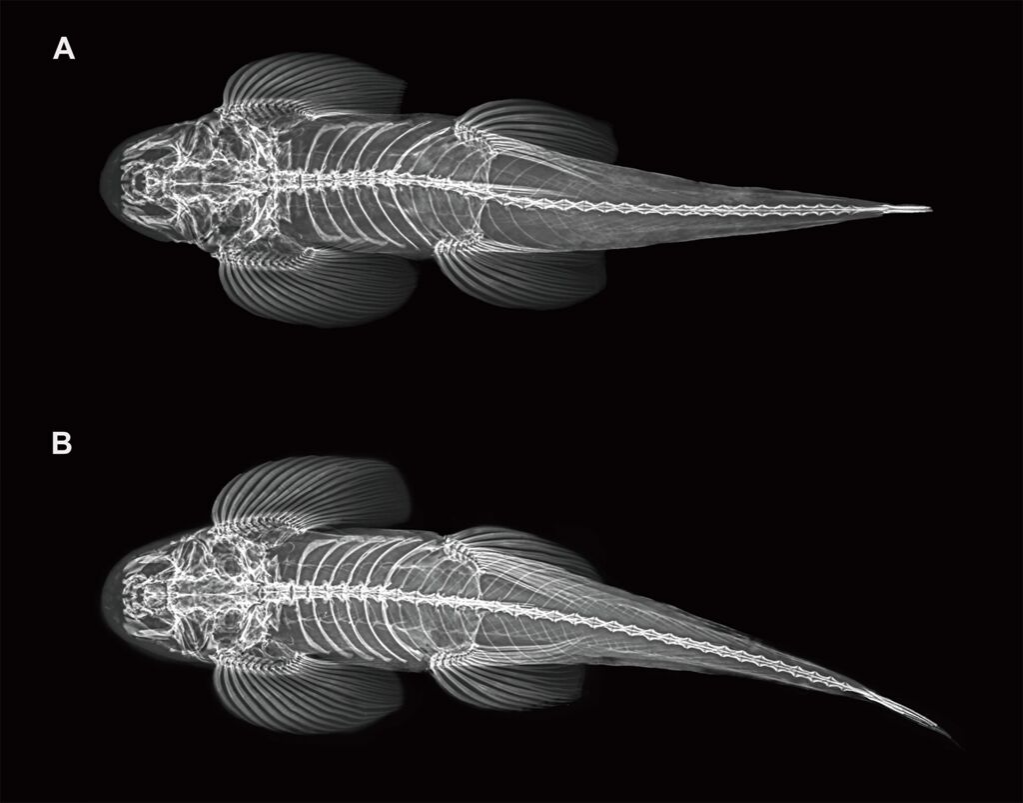
Dorsal profile of the skeleton of of *Balitorayingjiangensis* shown under X-ray; **A** KIZ1995000888 (Holotype); **B** KIZ1995000889 (Paratype).

Critical resolution emerged through examination of the Chinese Irrawaddy populations. Chen (2006) described *Hemimyzonyingjiangensis* from Yingjiang County (upper Irrawaddy drainage, China), classifying it under *Hemimyzon*, based on purported three simple pelvic rays. Our re-examination of type specimens and radiographic analysis (Fig. 6) revealed this was erroneous – specimens of *H.yingjiangensis* consistently presents two simple pelvic rays, aligning it with *Balitora* (Figs 3, 4, 6). Comprehensive morphological comparisons between the Irrawaddy (Myanmar and China) and Salween populations confirmed distinctiveness: Irrawaddy specimens differ consistently from both of the known species of the Salween, *B.burmanica* and *B.nantingensis* through meristic and proportional characters. Molecular phylogenetics strongly support this distinction, with Irrawaddy populations forming a monophyletic clade, sister to *B.burmanica* and *B.brucei* (Fig. 2). Genetic divergence between *B.yingjiangensis* and other congeners exceeds intraspecific variation, with the minimum interspecific p-distance (with *B.burmanica*) being 5.61%, significantly larger than maximum intraspecific p-distance (1.58%) (Table 4), confirming species-level differentiation. Thus, we validate *Balitorayingjiangensis* (Chen 2006) as the sole currently confirmed *Balitora* species in the Irrawaddy drainage, with previous *B.burmanica* records from this Basin representing misidentifications. The species' distribution range extends across the upper Irrawaddy system in both Chinese and Burmese sections. Despite all existing records of Balitorid fish from the Irrawaddy being confirmed as *B.yingjiangensis*, there might be a potential diversity of Balitoridae in the Irrawaddy River given that all neighbouring rivers are occupied by multiple Balitoridae species (Kottelat and Chu 1988, Chen and Tang 2000, Kottelat 2012), rendering a demand for comprehensive ichthylogical surveys on this diversity hotspot.

Notably, part of Kottelat's (Kottelat 1988) morphometrics overlap between *B.burmanica* and *B.yingjiangensis*, (such as CPL/CPD) suggesting possible historical specimen mixture. While *B.nantingensis* (endemic to the Nanting River, tributary of the Chinese Salween) shows superficial similarity to *B.burmanica*, insufficient material precludes definitive taxonomic assessment. The identity of *Balitora*. spp from the Karnaphuli River, the third river drainage where *B.burmanica* was originally recorded (Hora 1932) remains unresolved and requires separate investigation. This revision emphasises the need for integrated morphological-molecular approaches in clarifying *Balitora* diversity across southeast Asian River systems.

This re-description resolves a century-long confusion about *Balitora* distributions in the Irrawaddy Basin, establishing *B.yingjiangensis* as its endemic representative while restricting *B.burmanica* to Salween populations. Future studies should prioritise fresh material collection from Myanmar's upper Irrawaddy reaches to strengthen biogeographic interpretations.

### Phylogenetic research reveals defects of current morphological characteristics of Balitoridae

The non-monophyly of *Balitora*, *Hemimyzon*, *Sinogastromyzon* and *Lepturichthys* is confirmed in the phylogeny conducted in this study, which is similar to the results of previous studies (Tang et al. 2012, Luo et al. 2023). Non-monophyly of *Jinshaia* is new from this study, with *J.niulanjiangensis* being the sister group of *Lepturichthysfimbriata* instead of nesting with other two *Jinshaia* species, *J.sinensis* and *J.abbreviata*.

*Balitora* is divided into three monophyletic groups. Clade I or *Balitora* sensu stricto consists of all Indian and Indochinese species including *B.yingjiangensis*, characterised by possession of a single pair of maxillary barbels. Clade II consists only of *B.kwangsiensis*, the type species of *Sinohomaloptera*, distributed in the Pearl River, Red River and the Hainan Island. Clade III consists of two species with reduced eyes, namely *B.ludongensis* and *B.anlongensis*, distributed in limestone caves and adjacent water bodies from tributaries of the Pearl River (Fig. 2).

The non-monophyly of multiple genera in Balitoridae indicated that current intergeneric characteristics of Balitorids, namely the number of maxillary barbels, number of simple pelvic rays and the fusion of pelvic fins into a pelvic disc, could be result of convergent evolution given that most known balitorids are highly adapted to torrential habitats, thus unreliable in taxonomy. A comprehensive revision of Balitoridae, especially genera with complex oral structures, characterised by at least one row of papillae around mouth is needed. Sufficient specimens, morphological, skeletal X-ray photos and molecular statistics are required to complete this revision in the future.

The inconsistency of molecular markers in this study is inevitable due to unavailability of voucher specimens of several species, especially those not distributed in China. This practice should be avoided whenever possible in future studies.

## Supplementary Material

XML Treatment for Balitora yingjiangensis

## Figures and Tables

**Table 1.** Primers used in PCR amplification.

Gene	Primer	Sequences (5'-3')	Source
COI	Fish F1	TCAACCAACCACAAAGACATTGGCAC	Ward et al. (2005)
	Fish R1	TAGACTTCTGGGTGGCCAAAGAATCA	
Cyt *b*	L14724	GACTTGAAAAACCACCGTTG	Xiao et al. (2001)
	H15915	CTCCGATCTCCGGATTACAAGAC	

**Table 2.** Voucher code, sampling localities and accession numbers of all sequences used for molecular analysis. Newly-sequenced individuals and sequences from this study are highlighted in bold.

Number	Family/species	Locality (* type locality)	Voucher	Cyt *b*	COI
Nemacheilinae					
01	*Paracanthocobitismooreh*	Wynaad, KL, India	BNHS FWF 156	KT005604	KT005590
Balitoridae					
02	*Bhavaniaaustralis*	/	CBM:12692	NC_029440	NC_029440
03	*Homalopteraconfuzona*	/	CBM: ZF 11705	NC_033955	NC_033955
04	*Homalopteraocellata*	/	CBM: ZF 12287	NC_033953	NC_033953
05	*Homalopteraogilviei*	/	/	NC_031635	NC_031635
06	*Homalopteraparclitella*	/	/	NC_031634	NC_031634
07	*Pseudohomalopteraleonardi*	/	/	NC_008673	NC_008673
08	*Balitoraanlongensis*	Anlong County, Qianxinan Buyi and Miao Autonomous Prefecture, Guzihou Province, China	GZNU20230215020	OQ754143	OQ784689
09	*Balitoraanlongensis*	Anlong County, Qianxinan Buyi and Miao Autonomous Prefecture, Guzihou Province, China	GZNU20230215018	OQ754144	OQ784688
10	*Balitorabrucei*	/	CIFEFGB-Bb-02	MK732324	MK388804
11	*Balitorabrucei*	/	CIFEFGB-Bb-01	MK732323	/
12	***Balitoraburmanica***	**Yaeboat Dam, Hpa An, Myanmar**	**CXY20190208**	**PV819073**	**PV791992**
13	*Balitorachipkali*	Karnataka, Ramnagar,India	BNHS FWF 193	KU378016	KU378003
14	*Balitorachipkali*	Karnataka, Kamra, Joida, India	WILD-15-PIS-230	KU378017	KU378004
15	*Balitoraelongata*	Mengla County, Xishuangbanna Dai Autonomous Prefecture, Yunnan Province, China	IHB0301053	DQ105218	/
16	*Balitoraelongata*	Mengla County, Xishuangbanna Dai Autonomous Prefecture, Yunnan Province, China	IHB0301030	DQ105217	/
17	*Balitorakwangsiensis*	Yuanjiang, Yunnan Province, China	IHB0805546	JN177006	JN177071
18	*Balitorakwangsiensis*	Yuanjiang, Yunnan Province, China	IHB0805545	JN177004	/
19	*Balitoralaticauda*	Karnataka, Shimoga, India	WILD-15-PIS-232	KU378011	KU377998
20	*Balitoralaticauda*	Maharashtra, Ajara, India	WILD-14-PIS-070	KU378009	KU377996
21	*Balitoraludongensis*	Jingxi City, Baise City, Guangxi Zhuang Autonomous Region, China	GZNU20230215024	OQ754142	/
22	*Balitoraludongensis*	Jingxi City, Baise City, Guangxi Zhuang Autonomous Region, China	GZNU20230215023	OQ754141	/
23	*Balitoramysorensis*	Karnataka, Hattihole, India	BNHS: FWF 197	KU378019	KU378006
24	*Balitoramysorensis*	Karnataka, Hattihole, India	WILD-15-PIS-231	KU378018	KU378005
25	***Balitoratchangi***	**Weixi Lisu Autonomous County, Diqing Tibetan Autonomous Prefecture, Yunnan Province, China**	**WL20240228**	**PV819074**	**PV791993**
26	***Balitorayingjiangensis***	**Ruili City, Dehong Prefecture, Yunnan Province, China**	**KIZ20220332**	**PV819075**	**PV791994**
27	***Balitorayingjiangensis***	Nabang Town, Yingjiang County, Dehong Prefecture, Yunnan Province, China*	**KIZ20220255**	**PV819076**	**PV791995**
28	***Balitorayingjiangensis***	Nabang Town, Yingjiang County, Dehong Prefecture, Yunnan Province, China*	**KIZ20220256**	**PV819077**	**PV791996**
29	***Balitorayingjiangensis***	Nabang Town, Yingjiang County, Dehong Prefecture, Yunnan Province, China*	**ZYY20240048**	**PV819078**	**PV791997**
30	***Balitorayingjiangensis***	Nabang Town, Yingjiang County, Dehong Prefecture, Yunnan Province, China*	**KIZ20220257**	**PV819079**	**PV791998**
31	***Balitorayingjiangensis***	Nabang Town, Yingjiang County, Dehong Prefecture, Yunnan Province, China*	**ZYY20240049**	**PV819080**	**PV791999**
32	***Balitorayingjiangensis***	Nabang Town, Yingjiang County, Dehong Prefecture, Yunnan Province, China*	**KIZ20220258**	**PV819081**	**PV792000**
33	***Balitorayingjiangensis***	**Chan Khaung Stream, Putao District, Kachin State, Myanmar**	**CXY20150315**	**PV819082**	**PV792001**
34	***Balitorayingjiangensis***	**Stream near Hkyenhpa Populated place, Putao District, Kachin State, Myanmar**	**QT20170106**	**PV819083**	**PV792002**
35	***Balitorayingjiangensis***	**Stream near Hkyenhpa Populated place, Putao District, Kachin State, Myanmar**	**QT20170107**	**PV819084**	**PV792003**
36	***Balitorayingjiangensis***	**Stream near Hkyenhpa Populated place, Putao District, Kachin State, Myanmar**	**QT20170108**	**PV819085**	**PV792004**
37	***Balitorayingjiangensis***	**Stream near Hkyenhpa Populated place, Putao District, Kachin State, Myanmar**	**QT20170109**	**PV819086**	**PV792005**
38	***Balitorayingjiangensis***	**A primary tributary of Chindwin River, Yaw Su Village, Sagaing, Myanmar**	**CXY20190496**	**PV819087**	**PV792006**
39	*Hemimyzonformosanum*	/	Hf01	AY392475	/
40	***Hemimyzonmacroptera***	**Kaiyuan City, Honghe Hani Autonomous Prefecture, Yunnan Province, China**	**PXR20231113**	**PV819088**	**PV792007**
41	***Hemimyzonmegalopseos***	**Kaiyuan City, Honghe Hani Autonomous Prefecture, Yunnan Province, China**	**PXR20231011**	**PV819089**	**PV792008**
42	***Hemimyzonnujiangensis***	**Yongde County, Lincang City, Yunnan Province, China**	**ZYY20230068**	**PV819090**	**PV792009**
43	***Hemimyzonnujiangensis***	**Longling County, Lincang City, Yunnan Province, China**	**LSW202278**	**PV819091**	**PV792010**
44	*Hemimyzonyaotanensis*	Banan District, Chongqing City, China	IHB0809019	JN176994	/
45	***Jinshaiaabbreviata***	**Leshan City, Sichuan Province, China**	**PXR20240816**	**PV819092**	**PV792011**
46	***Jinshaianiulanjiangensis***	**Deze Township, Zhanyi District, Qujing City, Yunnan Province, China**	**LHT20240501**	**PV819093**	**PV792012**
47	*Jinshaiasinensis*	Qingjiang, Hubei Province, China	IHCAS0307118	DQ105210	/
48	*Lepturichthysdolichopterus*	/	LdHoun42	AY281279	/
49	*Lepturichthysfimbriata*	/	LfC17-1	AY281256	/
50	*Lepturichthysfimbriata*	Jiangxia District, Wuhan City, Hubei Province, China	IHB0809008	GU084243	JN177086
51	*Metahomalopteraomeiensis*	Hanyuan County, Ya’an City, Sichuan Province, China	IHB0000100	DQ111990	DQ112166
52	*Sinogastromyzonsichangensis*	/	IHB0301073	JN176999	JN177227
53	*Sinogastromyzonszechuanensis*	/	IHB0809029	JN176995	JN177110
54	*Sinogastromyzonszechuanensis*	Leshan city, Sichuan Province, China	IHCAS0307119	DQ105213	/
55	*Sinogastromyzontonkinensis*	Yuanjiang, Yunnan Province, China	IHB0805543	JN177002	JN177074
56	*Sinogastromyzontonkinensis*	Yuanjiang, Yunnan Province, China	IHB0805544	JN177003	JN177075

**Table 3.** Morphometric measurements of *Balitorayingjiangensis* compared with *B.nantingensis*, *B.burmanica* and *B.brucei*. Numbers in brackets indicate the number of specimens.

Characters	*B.yingjiangensis* (n = 11)				*B.burmanica* (n = 4)			*B.nantingensis* (n = 4)				*B.brucei* (Kottelat,1988)	
	Holotype	Range	Mean	S.D.	Range	Mean	S.D.		Range	Mean	S.D.	Range	Mean
Dorsal-fin rays	III+7	III+7	III+7		III+7-8	III+ 8			III+7	III+7		III+ 8½	
Pectoral-fin rays	Ⅸ+14	Ⅸ+14	Ⅸ+14		Ⅷ+13-10	Ⅷ+14			Ⅷ+14	Ⅷ+14		XI-X+10-12	
Pelvic-fin rays	Ⅱ+9	Ⅱ+9	Ⅱ+9		Ⅱ+8-9	Ⅱ+9			Ⅱ+9	Ⅱ+9		Ⅱ+9-10	
Anal-fin rays	III+5½	III+5½	III+5½		III+5	III+5			III+5	III+5		III+5½	
Caudal-fin rays	6+7	6+7	6+7		6+7	6+7			6+7	6+7			
Lateral-line pore/scales	63	67-63	65		64-62	63			62-59	61		66-61	63
Total length (mm)	88.56	102.83-37.61	80.43		80.38-46.25	63.97			74.63-68.22	71.63			
Standard length (mm)	79.68	87.34-32.63	68.32		64.89-38.20	53.1			62.84-56.44	58.48		105.60-64.30	
Head length (mm)	15.37	16.63-6.68	13.20		14.21-8.07	11.58			13.04-11.79	12.66			
Caudal peduncle length/depth	3.78	4.47-3.71	3.95	0.25	3.46-3.03	3.16	0.2		2.94-2.62	2.81	0.13	4.00-3.20	3.50
In percentage of SL													
Head length	19.29	20.47-18.35	19.46	0.75	22.24-21.12	21.76	0.46		23.10-20.54	21.66	1.26	22.0-18.50	20.00
Body depth	11.65	11.82-9.31	10.52	0.10	12.07-9.08	10.48	1.54		13.22-11.3	12.08	0.90	12.50-9.60	10.80
Body width	17.47	19.92-14.74	17.25	1.60	17.81-10.47	15.22	3.36		18.58-15.43	17.2	1.47	21.00-17.70	19.40
Dorsal-fin length	20.87	23.07-17.99	20.32	1.51	22.25-17.61	20.36	2.11		24.32-19.54	21.13	2.16	21.40-17.00	
Pectoral-fin length	27.06	29.43-21.51	25.61	2.19	26.41-21.62	23.81	2.05		24.44-22.26	23.71	0.98	30.60-26.80	
Pelvic-fin length	21.06	23.9-17.83	21.48	1.69	21.59-16.54	19.47	2.19		21.41-18.26	19.74	1.35	24.40-21.50	
Anal-fin length	12.61	14.83-11.23	12.84	1.21	13.33-8.89	11.75	1.99		13.97-11.3	12.23	1.19	12.70-9.70	
Dorsal-fin base length	12.76	16.84-12.00	13.58	1.42	13.61-10.38	12.64	1.52		15.6-13.68	14.19	0.94		
Anal-fin base length	9.36	9.96-5.10	7.37	1.46	7.09-5.22	6.19	0.89		7.73-5.64	6.88	1.00		
Predorsal length	42.57	46.85-36.13	42.00	2.63	49.39-42.01	44.50	3.34		45.37-41.86	43.62	1.51	46.30-43.20	44.70
Prepelvic length	41.84	47.90-39.08	43.05	2.68	47.30-41.71	44.61	2.39		47.37-42.89	45.54	2.01	46.60-42.20	44.60
Pre-anal length	73.57	79.25-66.31	74.16	3.67	77.64-73.34	76.10	1.89		81.19-74.8	77.36	2.83	82.40-72.0	76.30
Caudal peduncle length (CPL)	15.98	19.38-15.97	17.71	1.12	17.64-15.92	16.97	0.75		17.30-14.42	15.96	1.23	21.40-16.70	18.90
Caudal peduncle depth (CPD)	4.23	4.83-4.17	4.48	0.22	5.81-5.01	5.36	0.34		6.28-5.03	5.67	0.53	5.80-5.10	5.40
In percentage of HL													
Snout length	50.81	53.74-43.58	48.27	3.17	51.54-42.83	45.77	4.00		44.69-37.65	41.13	3.02		
Eye diameter	17.76	20.05-14.50	16.16	1.64	19.08-14.21	15.87	2.28		12.89-11.19	12.18	0.83		
Interorbital distance	41.57	43.87-37.77	41.37	1.69	37.02-34.13	35.98	1.26		40.62-31.28	36.1	3.83		
Maxillary barbel length	9.12	11.36-7.03	9.06	1.46	12.10-7.19	10.31	2.16		10.24-9.68	9.96	0.25		
Inner rostral barbel length	9.95	9.95-5.00	7.17	1.42	9.21-5.70	7.56	1.67		7.54-7.14	7.35	0.17		
Outer rostral barbel length	9.24	11.22-8.16	9.42	1.01	11.61-7.60	10.00	1.06		11.28-7.68	9.77	1.58		
Mouth width	22.06	25.30-20.17	22.17	1.61	25.71-17.31	20.39	3.69		19.33-14.87	17.11	2.37		

**Table 4.** Uncorrected p-distance (%) amongst eight *Balitora* spp., based on mitochondrial Cyt *b* gene. "/" is for maximum intraspecific p-distance of species represented by only one individidual or sequences of all individuals are identical.

	*Balitorayingjiangensis*	*Balitoraburmanica*	*Balitoraelongata*	*Balitoratchangi*	*Balitorabrucei*	*Balitorachipkali*	*Balitoramysorensis*	*Balitoralaticauda*
*Balitorayingjiangensis*	1.68							
*Balitoraburmanica*	5.61	/						
*Balitoraelongata*	8.33	8.97	1.15					
*Balitoratchangi*	7.85	7.53	10.10	/				
*Balitorabrucei*	8.49	7.05	10.74	9.13	/			
*Balitorachipkali*	10.74	9.78	11.06	9.62	9.94	0.09		
*Balitoramysorensis*	8.97	8.81	10.58	9.78	8.97	11.70	/	
*Balitoralaticauda*	9.46	8.33	10.58	9.29	9.13	3.69	10.42	0.45
